# Combination of Rotte Y Applicator and Standard Tandem for Medically Inoperable Endometrial Cancer

**DOI:** 10.1016/j.adro.2021.100787

**Published:** 2021-09-11

**Authors:** Yoshiaki Takagawa, Sachiko Izumi, Tomoyuki Okano, Eiichi Takahashi, Yuki Wakamatsu, Midori Kita

**Affiliations:** a Department of Radiation Oncology, Southern Tohoku Proton Therapy Center, Fukushima, Japan; b Department of Radiology, Tokyo Metropolitan Tama Medical Center, Tokyo, Japan

## Introduction

Endometrial cancer (EC) is the most common gynecologic malignancy in developed countries; it is the fourth leading cause of cancer and the sixth leading cause of cancer-related deaths among women in the United States.^1^ Morbidity due to EC continues to rise worldwide due to the global prevalence of obesity and the aging of populations.^2^ Surgery is the standard initial treatment for most patients with EC. Definitive radiation therapy (RT), with or without chemotherapy, may be offered to patients with medically inoperable EC. For these patients, RT is performed using external beam radiation therapy (EBRT) and/or brachytherapy (BT). Unlike the cervix, the uterine body has a large lumen; therefore, a modified Heyman packing method and various intracavitary applicators have been developed for BT for EC treatment. Currently, one of the standard applicators used for administering BT to patients with EC is the Rotte “Y” applicator. However, we often experience that for a large uterine body, the dosimetric coverage achieved through using the Rotte Y applicator for BT is not satisfactory. There are few reports regarding the use of 3-channel BT applicators for patients with EC. Moreover, with respect to gastrointestinal toxicity, attention must be paid to the sigmoid colon as it is closely related to the uterine body.

Herein, we report a case of a patient with medically inoperable EC who was treated with a novel technique of combining the Rotte Y applicator and standard tandem (3-channel) BT.

## Case Presentation

**Fig. 1:**
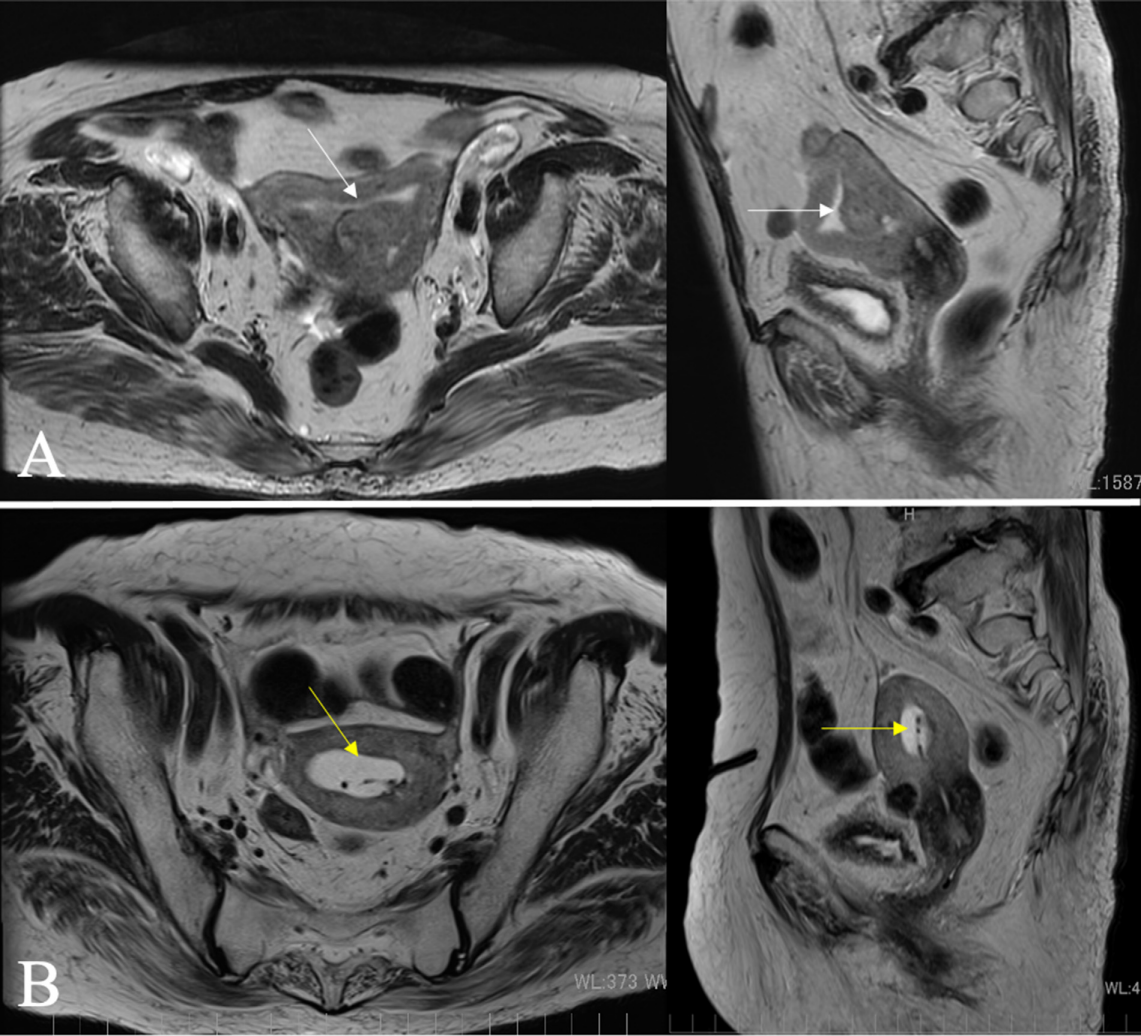
(a) Pretreatment magnetic resonance imaging (MRI) results revealed a tumor in the posterior wall of the uterine body (white arrow). (b) Pelvic MRI revealed a complete response for the tumor (yellow arrow) at 2 months after radiation therapy (the intrauterine device remained in place).

An 84-year-old woman received a diagnosis of endometrial clear cell carcinoma. Computed tomography (CT) and magnetic resonance imaging (MRI) were performed. CT revealed multiple enlarged lymph nodes in the pelvis and para-aortic area. The pretreatment MRI showed a 3-cm tumor in the posterior wall of the uterine body (Fig 1A). The clinical stage of the EC was T1bN2M0, and the International Federation of Gynecology and Obstetrics stage was IIIC2. Because of advanced age, chronic renal failure, and lower performance status, definitive RT as monotherapy was planned. She had placed intrauterine device (IUD) decades ago and it remained still. Factors affecting the implant included pre-existing IUD. Removal of the IUD was attempted but unsuccessful.

**Fig. 2:**
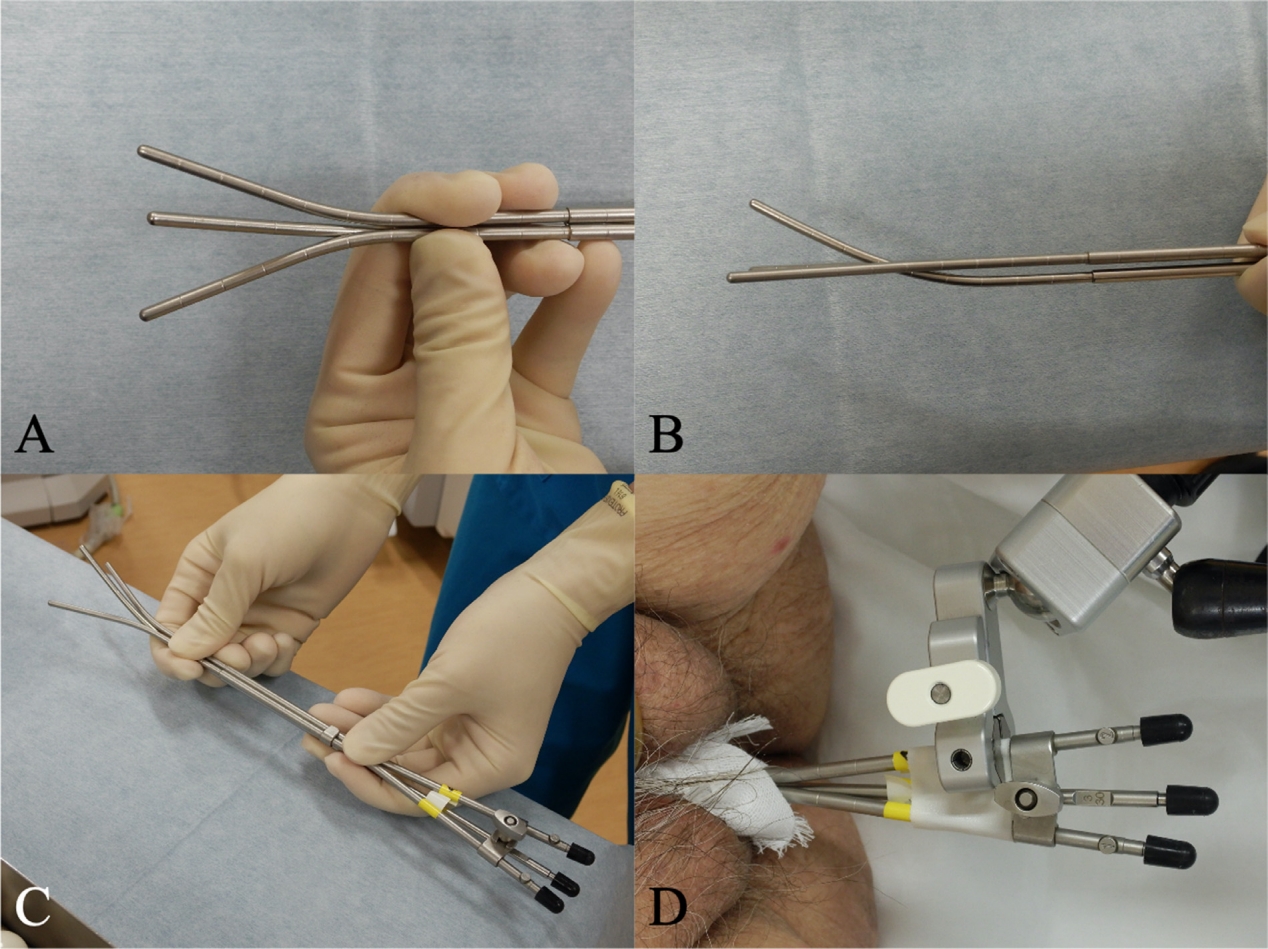
Photograph of the Rotte Y applicator combined with a Fletcher intrauterine 30° tandem. (a) Front view and (b) side view of the tips of the combined applicators. (c) Overall image of the combined applicators. (d) Image of the immobilization of the combined applicators during brachytherapy.

**Fig. 3:**
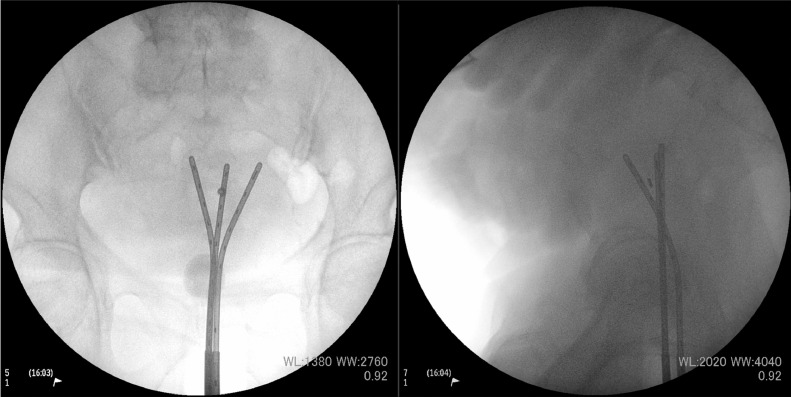
Anteroposterior and lateral fluoroscopic x-ray images of the combination of the Rotte Y applicator and a Fletcher intrauterine 30° tandem during brachytherapy.

**Fig. 4:**
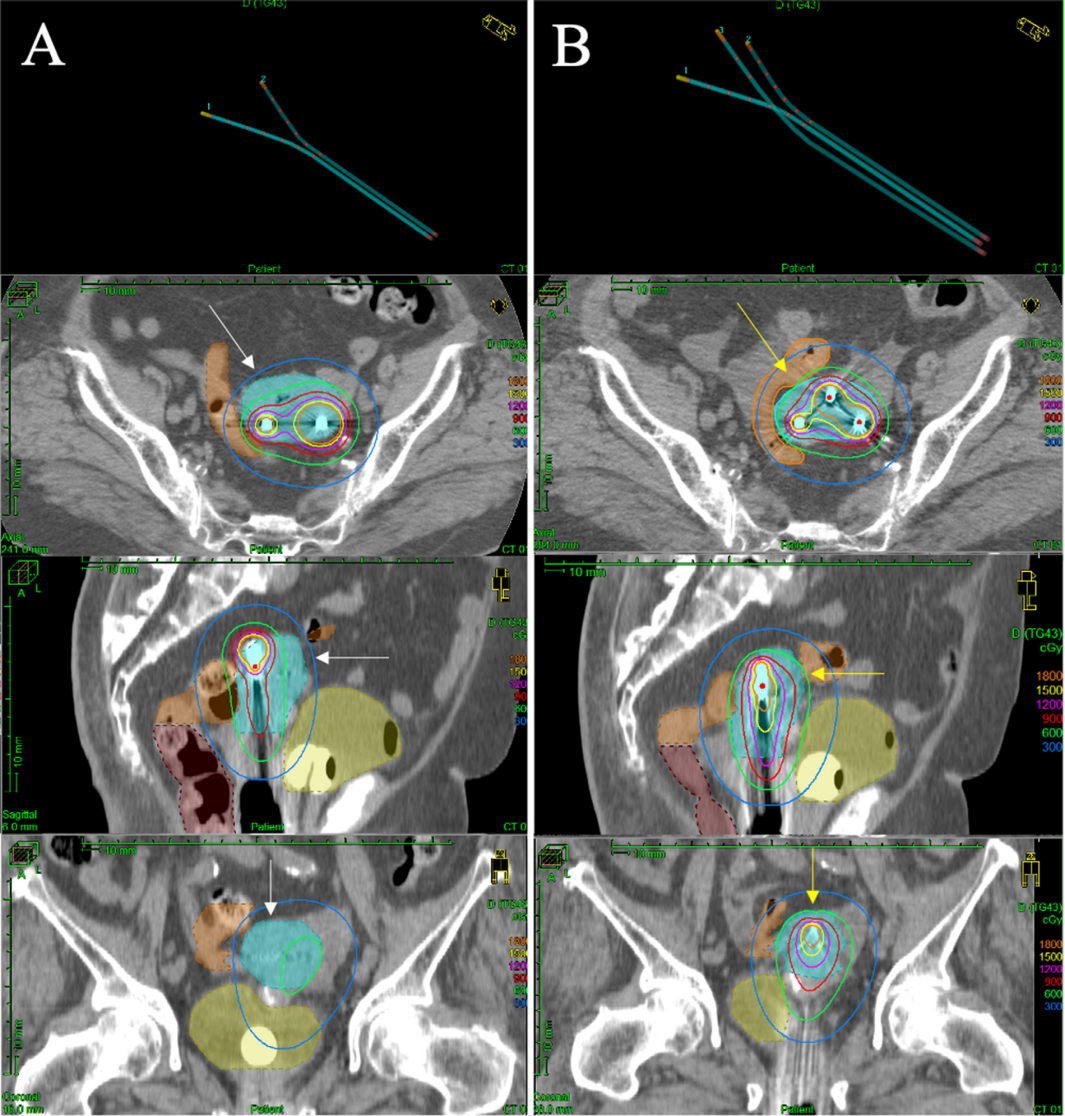
Comparison of dose distribution in image guided brachytherapy. (a) Treatment with the Rotte Y applicator alone (conventional session). (b) Treatment with the Rotte Y applicator and a tandem (hybrid session). Light blue shade, the clinical target volume (CTV); yellow shade, the bladder; brown shade, the rectum; orange shade, the sigmoid colon. The green line is the 100% (6 Gy) isodose line.

After consultation with the radiation oncology department, EBRT followed by BT was planned. The EBRT technique was 3-dimensional (3D) conventional RT because our linear accelerator did not support pelvic irradiation using the intensity modulated radiation therapy technique. We set the external beam radiation field, which included the whole pelvis and para-aortic area. The EBRT dose was 50.4 Gy, delivered in 28 fractions. A midline block (3-cm width at the isocenter) was inserted into the treatment field after delivering 30.6 Gy in 17 fractions to the whole pelvis. After 30.6 Gy irradiation, we administered definitive BT with the Rotte Y applicator (Elekta, Sweden) weekly. For remote afterloading, we used Ir-192 with the microSelectron Digital (HDR-V3) afterloader and the Oncentra Brachy treatment planning system/module (Elekta, Sweden). We performed CT-based, image guided brachytherapy in every BT session. The BT dose was 24 Gy delivered in 4 fractions to the uterine body as the clinical target volume (CTV). We did not include the vagina in the radiation field of BT because of the tumor location, the patient's age, and possible toxicity. For BT, we aimed for a 100% prescription isodose line to fit the CTV (point prescription was not used). On the day before every BT session, we inserted laminaria into the cervical canal for cervical dilation. During applicator implantation, anesthesia was induced using intravenous injection of pentazocine and hydroxyzine hydrochloride. To immobilize the applicator, we performed vaginal gauze packing in every BT session. During the first and second BT sessions, we only used the Rotte Y applicator (conventional sessions). Before every insertion of the applicator, we dilated the cervix with a Hegar dilator. The interval of the tip of the Rotte Y applicator was 40 mm. However, the average CTV D90, D98, and V100 values for the conventional sessions were 5.7 Gy, 4.5 Gy, and 87.14%, respectively (Table 1), and these were insufficient for definitive therapy. Therefore, for the third and fourth BT sessions, we combined the Rotte Y applicator with a Fletcher 30° standard tandem (Elekta, Sweden) (hybrid sessions). We set the tandem under the Rotte Y applicator to fit in the groove of the Rotte Y applicator (Fig 2). The tips of the applicators were triangular. After applicator insertion, adding standard vaginal gauze packing, we immobilized these applicators by taping. The anteroposterior and lateral fluoroscopic x-ray images of the Rotte Y applicator and tandem combination during BT are shown in Figure 3. The dosimetric coverage of the CTV in the hybrid sessions was significantly improved (Fig 4). The average CTV D90, D98, and V100 values for the hybrid sessions were 7.4 Gy, 5.5 Gy, and 96.9%, respectively (Table 1). The total biologically equivalent dose in 2 Gy fractions (Gy_EQD2_) of EBRT (30.6 Gy/17 fractions) plus BT (24 Gy/4 fractions), based on the linear-quadratic model for CTV D90, was 66.5 Gy_EQD2_, assuming an α/β ratio of 10. For the organs at risk (OAR), the Gy_EQD2_ was assumed to have an α/β ratio of 3. The total Gy_EQD2_ of EBRT plus BT for the rectum, bladder, and sigmoid colon were 65.7, 46.8, and 83.6 Gy_EQD2_, respectively. The hybrid sessions could provide a higher dose to the CTV and a lower dose to the OAR, especially for the sigmoid colon, than the conventional sessions (Table 1).

**Table 1:** Dosimetric parameters of each brachytherapy

Brachytherapy	CTV volume (cm^3^)	CTV D90 (Gy)	CTV D98 (Gy)	CTV V100 (%)	Rectum D2cc (Gy)	Bladder D2cc (Gy)	Sigmoid D2cc (Gy)
# 1 (conventional)	77.72	5.2	4.1	83.36	2.5	4.4	6.7
# 2 (conventional)	99.08	6.1	5.0	90.92	4.0	5.8	7.7
# 3 (hybrid)	84.01	7.4	5.0	95.32	3.9	5.3	6.7
# 4 (hybrid)	82.62	7.5	6.0	98.01	2.2	5.5	5.9

*Abbreviations:* CTV = clinical target volume; D2cc = doses for most exposed 2 cc volumes; Dn = minimal dose delivered to n% of target volume; V100 = fractional volume of the organ receiving 100% of the prescribed dose.

Two months after RT, the endometrial tumor had regressed, as revealed through pelvic MRI results (Fig 1b), and this was considered a complete response. There were still multiple enlarged pelvic lymph nodes, and reactive changes were diagnosed. Ten months after RT, there was no recurrence of EC. There were no severe acute or late toxicities of RT during follow-up.

## Discussion

The standard initial treatment for patients with early-stage EC is surgery. However, due to the increasing aging population and the prevalence of obesity, the number of patients with medically inoperable EC with high-risk factors for surgery has also increased. RT showed high local control rates and improved survival in older adults with medically inoperable EC of stages I to II.^3^

In 1941, Heyman et al^4^ advocated using a packing method in BT for patients with EC. Since 1993, a modern, high-dose rate remote afterloading system has been used, and a modified Heyman packing method has been developed.^5^ However, using this method to treat Asian women with smaller uteri than Western women is difficult; therefore, the packing method is not popular in Asia. The Rotte Y applicator is one of the standard applicators used to administer BT for patients with EC. Coon et al^6^ demonstrated 10-year results of using the Rotte Y applicator for the definitive treatment of patients with medically inoperable EC. They treated 49 patients with medically inoperable EC using 2-dimensional (2D) and 3D BT planning. During the median follow-up time of 33 months, the 3- and 5-year cause-specific survival rates were 93% and 87%, respectively. They also reported that late grade ≥ 2 toxicity at 3 and 5 years was 13%. On the other hand, we must always pay attention to the sigmoid colon as it is closely related to the uterine body receiving BT for EC. Beriwal et al^7^ reported comparing 2D versus 3D dosimetry for Rotte Y applicator high-dose rate BT for medically inoperable patients with EC. In their study, they mentioned that the dose to the sigmoid colon was high, and attention should be paid to the sigmoid dose at the time of treatment planning. In the present case, we could not deliver a higher dose to the CTV with only the Rotte Y applicator because of the sigmoid colon (Fig 4a).

**Fig. 5:**
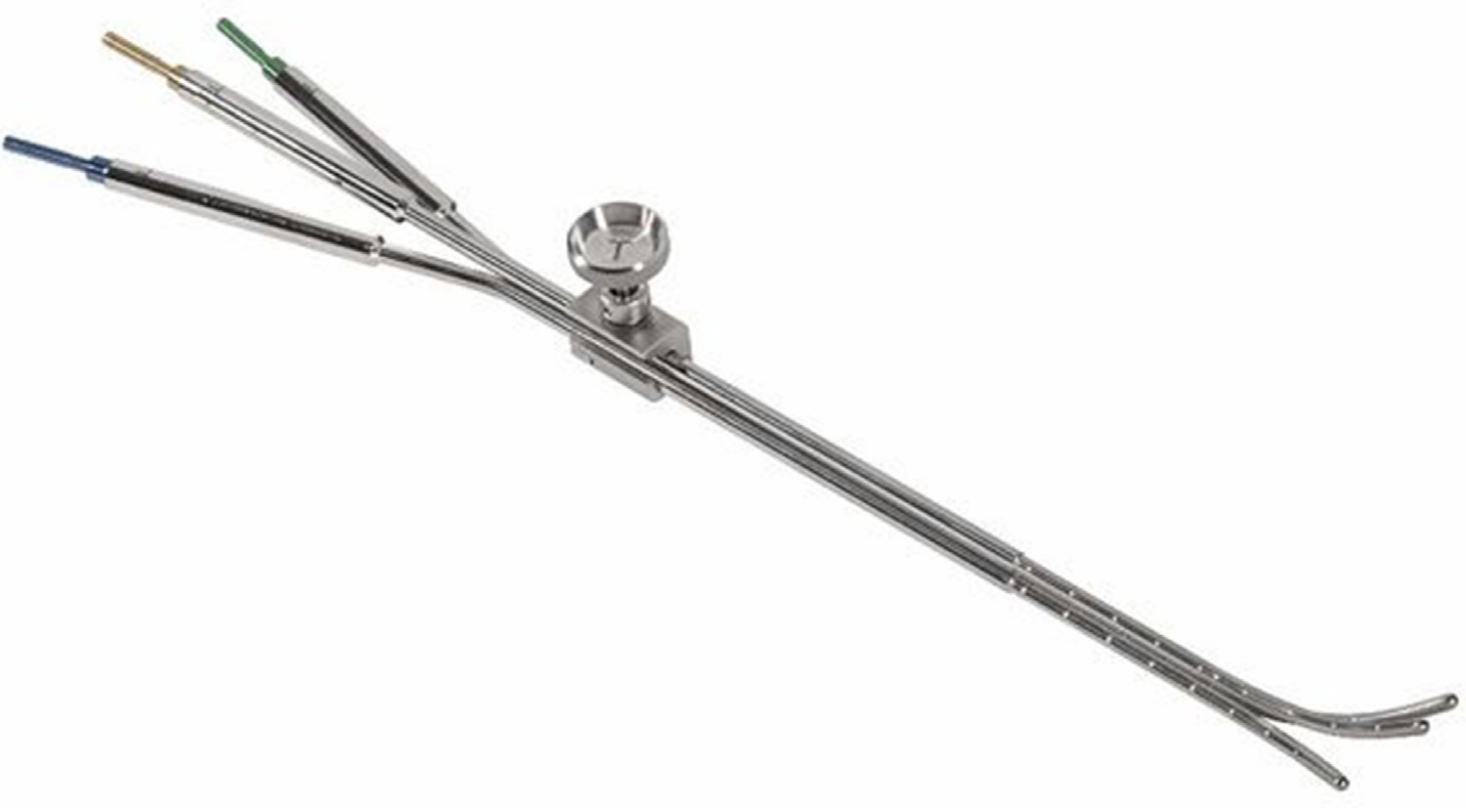
Image of the commercially available 3-channel endometrial applicator.^10^

Nori et al^8^ and Inoue et al^9^ reported using a 3-channel intracavitary applicator in BT for patients with EC. In both cases, by the 3-channel applicator, a good dose distribution was achieved for the uterine body. Nowadays, the 3-channel endometrial applicator (Mick Radio-Nuclear Instruments, Inc, Mount Vernon, NY) is commercially available (Fig 5).^10^ Johnson et al^11^ published a study of single, dual, and triple tandem applicators to treat intact uterine cancer using this 3-channel endometrial applicator. In the 18 treatment plans of the 3 patients, they mentioned that the triple tandem applicator achieved better uterus coverage in the selected patient than either single or dual tandem applicators.

In the present case, an original 3-channel applicator was easily developed by combining the Rotte Y applicator and Fletcher standard tandem. Using this original 3-channel applicator, dosimetric improvement was achieved for the large uterine body, without an increase in the dose delivered to the sigmoid colon to an amount more than that delivered when only using the Rotte Y applicator (Fig 4b). For selective patients with medically inoperable EC who have a large uterine body, 3-channel BT may have better dosimetric coverage for the uterus with less toxicity of OARs than conventional Rotte Y applicator BT. Therefore, our novel and simple technique can present the option of a 3-channel BT for EC treatment for facilities that do not have a commercially available 3-channel endometrial applicator.

However, to insert the combined Rotte Y applicator and tandem, sufficient cervical dilation is necessary. This technique is not suitable for patients with EC who have narrow cervical canals. When performing this technique, one must be careful of the possibility of uterine perforation, especially when treating older adult patients with EC who have thin uterine walls.

## Conclusions

Our novel and simple technique, the combination of Rotte Y applicator and standard tandem, is a good 3-channel BT option for patients with medically inoperable EC with large uteri.
